# Repetitive transcranial magnetic stimulation for apathy in patients with neurodegenerative conditions, cognitive impairment, stroke, and traumatic brain injury: a systematic review

**DOI:** 10.3389/fpsyt.2023.1259481

**Published:** 2023-11-15

**Authors:** Adrian I. Espiritu, Takatoshi Hara, Joy Kirsten Tolledo, Mervin Blair, Amer M. Burhan

**Affiliations:** ^1^Ontario Shores Centre for Mental Health Sciences, Whitby, ON, Canada; ^2^Department of Psychiatry and Department of Medicine, Division of Neurology, University of Toronto, Toronto, ON, Canada; ^3^Department of Clinical Epidemiology, College of Medicine, University of the Philippines Manila, Manila, Philippines; ^4^Department of Rehabilitation Medicine, National Center Hospital, National Center of Neurology and Psychiatry, Tokyo, Japan; ^5^Department of Rehabilitation Medicine, The Jikei University School of Medicine, Tokyo, Japan; ^6^Lawson Research Institute, London, ON, Canada; ^7^Department of Psychiatry, Temerty Faculty of Medicine, University of Toronto, Toronto, ON, Canada

**Keywords:** repetitive transcranial magnetic stimulation, non-invasive brain stimulation, apathy, neurodegeneration, cognitive impairment, stroke, traumatic brain injury, systematic review

## Abstract

**Background:**

We aimed to determine the effects and tolerability of repetitive transcranial magnetic stimulation (rTMS) on apathy in patients with neurodegenerative conditions, mild cognitive impairment (MCI), stroke, and traumatic brain injury (TBI) via systematic review.

**Methods:**

We conducted a systematic search in major electronic health databases, including PubMed, Scopus, and PsycINFO, covering the period from inception to June 2023. Comparative clinical trials and cohort studies, and studies with before-after designs were considered for inclusion. We used the Cochrane Risk of Bias and the National Institutes of Health (NIH) tools to assess methodological quality.

**Results:**

Out of 258 records identified, 14 studies met our eligibility criteria (11 randomized controlled trials (RCT) and 3 studies utilized before-and-after designs) with a total of 418 patients (overall female-to-male ratio 1:1.17) included in the review. The overall methodological quality of the included studies was assessed to be fair to good. The stimulation parameters used varied considerably across the studies. The summary findings of our review indicate the following observations on the effects of rTMS on apathy: (1) the results of all included studies in Alzheimer’s disease investigating the effects of rTMS on apathy have consistently shown a positive impact on apathy; (2) the majority of studies conducted in Parkinson’s disease have not found statistically significant results; (3) a single study (RCT) on patients with primary progressive aphasia demonstrated significant beneficial effects of rTMS on apathy; (4) the trials conducted on individuals with MCI yielded varying conclusions; (5) one study (RCT) in chronic stroke suggested that rTMS might have the potential to improve apathy; (6) one study conducted on individuals with mild TBI did not find a significant favorable association on apathy; and (7) the use of different rTMS protocols on the populations described is generally safe.

**Conclusion:**

The feasibility of utilizing rTMS as a treatment for apathy has been suggested in this review. Overall, limited evidence suggests that rTMS intervention may have the potential to modify apathy among patients with AD, PPA, MCI and chronic stroke, but less so in PD and mild TBI. These findings require confirmation by larger, well-designed clinical trials.

## 1. Introduction

Apathy is one of the clinical features in various neuropsychiatric disorders that may significantly interfere with rehabilitation efforts toward independent living and social participation. Apathy is manifested by a deficiency in goal-directed activities or motivation and blunted emotional responsiveness and spontaneity, which could manifest on a spectrum depending on the underlying disorder, severity, and apathy dimension involved (i.e., behavioral, cognitive, or affective) (1, 2). The frequency of apathy tends to increase with age, particularly in individuals aged 65 and above (3).

The impact of apathy can be variable, but often time it hinders the individual’s ability to benefit from rehabilitation efforts. For instance, a study examined the functional improvement of spontaneity post-stroke found that the group with apathy showed less improvement on the Barthel Index compared to those without apathy, suggesting that apathy is a major obstacle in the field of cognitive rehabilitation, and may affect subsequent clinical outcomes (4). Moreover, a large population-based longitudinal study (*n* = 3,626) reported that cognitively impaired geriatric population with apathy are at 3.1-fold increased risk of one-year mortality compared to their counterparts without apathy (5). Overall, apathy represents a complex and multifaceted symptom that has far-reaching implications. Its diverse dimensions and associations with functional decline and mortality, as well as caregiver burden and increased healthcare costs, emphasize the importance of understanding and addressing apathy in clinical practice and research (6, 7).

The current range of pharmacological interventions for apathy is restricted and a proportion of individuals may experience intolerance precluding their use. Methylphenidate, a dopaminergic agent, has been observed to yield modest benefits in improving apathy (8–11). Cholinergic and glutamatergic drugs, serotonin reuptake inhibitors, several behavioral approaches and other non-pharmacological interventions were previously investigated on apathy with variable and limited effects (2, 12, 13). The lack of approved effective treatment for apathy, necessitates the exploration of alternative approaches to effectively address this debilitating symptom.

Recently, the role of noninvasive brain stimulation (NIBS) as a method that can induce excitatory or inhibitory changes in the underlying cerebral cortex in a nonintrusive manner and potentially induce long-term neuroplastic changes has received a great deal of attention (14, 15). In general, NIBS techniques use electrical and/or magnetic energy to modulate excitability in the underlying cerebral cortex in a non-invasive manner. Specifically, repetitive transcranial magnetic stimulation (rTMS) produces a time-varying magnetic field that runs perpendicular to the stimulation coil, which induces electric currents in the underlying cortical tissue that are nearly parallel to the coil. Different stimulation frequencies have different effects on cortical activity (1): high-frequency ( ≥ 5 Hz) stimulation enhances cortical activity (2); and low-frequency ( ≤ 1 Hz) stimulation exhibits inhibitory effects (14–16).

rTMS has known applications in the field of psychiatric disorders, particularly in the treatment of treatment-resistant depression (17, 18). Recently, it has been reported that NIBS may have therapeutic effects on cognitive function (19, 20). Our previous systematic reviews suggested that NIBS for stroke and head injury may show a certain degree of effect on cognitive function (20, 21). However, no thorough systematic reviews have been conducted of rTMS’ impact on apathy in people with neurodegenerative conditions, mild cognitive impairment (MCI), stroke or traumatic brain injury (TBI). Therefore, the aim of this study is to conduct a systematic review of the effect and safety of rTMS on apathy in these populations.

Our rationale for including various neurocognitive disorders is to explore the feasibility of utilizing the rTMS intervention in a number of neurocognitive disorders. Since the mechanisms of action of different NIBS modalities for these disorders may be distinct, we concentrated our investigation on the effects of rTMS in order to give a more in-depth analysis of the studies that utilized this intervention while also providing a clearer scope of the review.

## 2. Methods

### 2.1. Criteria for the selection of studies

We considered including randomized controlled trials (RCT) as well as prospective or retrospective cohort studies that incorporated a control group. Furthermore, we considered research that utilized a single-group, pre-post design. Investigations utilizing alternative/less rigorous research methodologies were eliminated from consideration. Human studies that used rTMS as an intervention (at least 5 consecutive sessions administered) for the improvement of apathy symptom and compared its effects to those of sham therapy, medication-only, no-treatment control, or to baseline measures were taken into consideration for inclusion. We covered populations with neurodegenerative disorders, cognitive impairment, stroke, or traumatic brain injuries in this review, regardless of age, sex, social construct of race/ethnicity, comorbidities or medications taken. We excluded studies that relied on secondary data or lacked complete accessibility to full-text reports. We also removed replicated reports, abstract or poster-only publications, outcomes lacking a corresponding description of the background and methods, animal studies, and reviews.

### 2.2. Outcome measures considered

The present review centered on the impact of rTMS on apathy within the aforementioned populations. We considered apathy as a condition presenting as a diminished or absent motivation that cannot be attributed to a decline in consciousness, cognitive impairment, or emotional distress, caused by observable alterations in affect, behavior, and cognition (1). To quantify this complex and multidimentional symptomatology, we took into account all studies that utilized any validated or standardized apathy scales or subscales that are commonly employed in clinical or research environments, as follows: Apathy Evaluation Scale (AES) (1, 22); Apathy Scale (AS) (23); Neuropsychiatric Inventory-Apathy subscale (NPI-AS) (24); Lille Apathy Rating Scale (LARS) (25); Apathy Inventory (26); Dementia Apathy Interview and Rating Scale (DAIR) (27); Apathy Scale for Institutionalized Patients with Dementia Nursing Home version (APADEM-NH) (28); Person-Environment Apathy Rating (PEAR) (29); Nonmotor Symptoms Questionnaire–Apathy subscore (NMSQ-AS) (30); and Dimensional Apathy Scale (DAS) (31). In addition to the apathy outcome scales, data pertaining to any adverse events reported in the studies that were included in the analysis were also gathered.

### 2.3. Search methods for the identification and the selection process of studies

The databases of MEDLINE by PubMed, Scopus and PsycINFO were searched to include studies from inception until April 2023 for potentially relevant records. Given our objective to conduct a comprehensive review to identify relevant studies that may be associated with the concept of apathy, we incorporated search terms that pertain to various dysexecutive syndromes, including anhedonia, abulia, akinetic mutism, among others. The applied search terms were as follows: ((neurodegenerative OR neurodegeneration OR “neurologic degenerative”) OR (“traumatic brain injury” OR “brain injury” OR “brain concussion” OR “cerebral concussion” OR “concussion” OR “traumatic encephalopathy”) OR (stroke OR “cerebral vascular accident” OR “cerebrovascular accident” OR “cerebral vascular disease” OR “cerebrovascular disease” OR “ischemic stroke” OR “ischaemic stroke” OR “hemorrhagic stroke”)) AND ((“non-invasive brain stimulation” OR “noninvasive brain stimulation” OR “NIBS”) OR (“transcranial magnetic stimulation” OR “repetitive transcranial magnetic stimulation” OR “rTMS” or “TMS”)) AND (apathy OR anhedonia OR abulia OR “akinetic mutism” OR hypoactivation OR “psychomotor retardation” OR “executive dysfunction” OR “executive disorder”).

The process of selecting studies was carried out utilizing the Covidence platform. Two reviewers (AIE and TH) conducted an independent assessment of the titles and abstracts of all records retrieved, utilizing the screening criteria. The full texts of all pertinent trials that met the screening criteria were obtained and evaluated by 2 reviewers (AIE and TH) based on the eligibility criteria. In the event of inconsistencies in meeting the screening and eligibility criteria, a third reviewer (MB) was consulted for further discussion. All studies that fulfilled the eligibility criteria were included in this review.

### 2.4. Assessment of risk of bias, data collection and analysis

The risk of bias in the included studies was assessed by 2 reviewers (AIE and TH), with a third reviewer (MB) serving as an arbitrator in the event of any discrepancies. The Risk of Bias (RoB) Tool 1 developed by Cochrane was employed for evaluating randomized controlled trials (RCTs) (32) while the National Institutes of Health (NIH) tool was employed to evaluate the quality of before-after studies that lacked a control group (33).

Two reviewers (AIE and JKT) independently conducted the data extraction process. The obtained data encompassed various aspects such as the study design, patients’ characteristics, trial settings, treatment and comparator regimen features, and pertinent outcomes determined by various apathy scales and detecting any adverse events. We gathered data on the incidence of variables of interest among participants, particularly the number of patients who were positive on a dichotomous variable and the total number of individuals in each group. We also collected relevant available data on important continuous variables, including mean/median pretreatment/posttreatment values and mean differences, and measures of dispersion such as standard deviations (SD)/standard error (SE)/95% confidence intervals (CI), and the number of patients in each treatment group. The presentations and analyzes of all data were done qualitatively.

## 3. Results

### 3.1. Included studies and population characteristics

**Figure 1:**
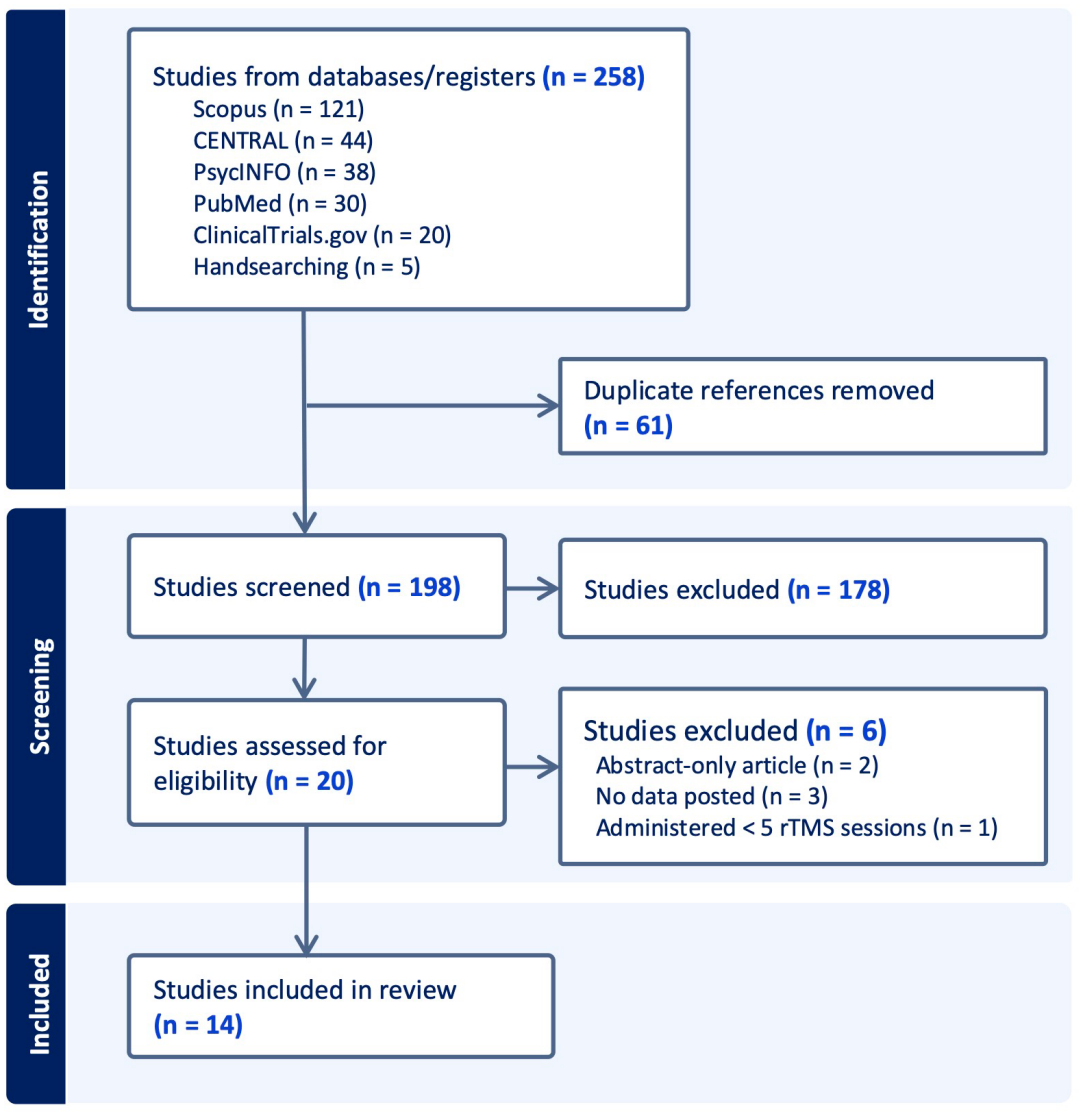
PRISMA flow diagram of information.

Figure 1 illustrates the PRISMA flow of information framework. A total of 258 records were identified from electronic databases and 5 from handsearching. Sixty-one duplicates were discarded. Out of 198 records screened, 178 were excluded on the basis of obvious irrelevancy and the rest were assessed for eligibility. Five studies were excluded due to the following reasons: abstract-only articles (2 studies); no available data (3 studies); and < 5 rTMS sessions administered (1 study). Finally, a total of 14 studies were included for qualitative analyzes in this review (see Table 1). Among these, there were 11 RCTs (34–44) and 3 studies that utilized a single-group, before-after design (45–47). One study (NCT00955032, labeled “ReStore” Study) was obtained from clinicaltrials.gov website which was unpublished; the narrative results were found from the funding agency website (39, 48). Three studies were participant-, study personnel (rTMS personnel)-, and outcome assessor-blinded (34, 40, 41), 6 studies were participant- and outcome assessor-blinded (35, 37, 39, 42–44), one study was participant-blinded only (36), and 1 trial did not specify their blinding methods (38). The duration of the follow-up period varies significantly, ranging from a minimum of 5 days to a maximum of 4 years.

**Table 1:** Characteristics of the included studies.

Study name	Study design	Intervention group	Comparison	Apathy outcomes measured	Sample size (n)	Length of follow-up	Country of enrollment	Enrollment period	Hospital/clinic of enrollment
Alzheimer’s disease									
Hu et al. (38)	Randomized controlled trial	(1) HF rTMS + tDCS (2) HF rTMS only	(1) tDCS only (2) Sham	Neuropsychiatric Inventory - Apathy subscale (NPI-AS)	All: 84 rTMS+tDCS: 21 rTMS only: 21 tDCS only: 21 sham: 21	12 weeks	China	NR	Department of Neurology of Xuanwu Hospital
Moreno et al. (47)	Single group before-after design study	NeuroAD procedure (HF rTMS + cognitive training)	Pretreatment measures	Apathy Inventory (AI)	All: 30	1.5 to 4 years	France	January 2015 February 2019	Clinique Bretéché
Nguyen et al. (46)	Single group before-after design study	NeuroAD procedure (HF rTMS + cognitive training)	Pretreatment measures	Apathy Inventory (AI)	All: 10	6 months	France	February 2015 to September 2015	NR
Padala et al. (41)	Randomized, participant-, study-personnel-, and outcome assessor-blinded, controlled trial	HF rTMS	Sham	Apathy Evaluation Scale-Clinician version (AES-C)	All: 20 rTMS: 9 sham: 11	12 weeks	USA	May 2014 to July 2019	Central Arkansas Veterans Healthcare System
Parkinson’s disease									
Brys et al. (34)	Randomized, participant-, study personnel-, and outcome assessor-blinded, controlled trial	HF rTMS	Sham	Apathy Evaluation Scale (AES)	All: 61 M1 rTMS and DLPFC rTMS: 20 M1 rTMS and DLPFC sham: 14 M1 sham and DLPFC rTMS: 12 M1 sham and DLPFC sham: 15	6 months	USA & Canada	May 2011 to June 2014	Beth Israel Deaconess Medical Center, New York University School of Medicine, Toronto Western Research Institute, University of California School of Medicine, Cleveland Clinic, University of Florida and University of North Dakota School of Medicine
NCT00955032	Randomized, participant- and outcome assessor-blinded, controlled trial	HF rTMS	Sham	Apathy Evaluation Scale (AES); Lille Apathy Rating Scale (LARS)	All: 24 rTMS: 16 Sham: 8	10 days	USA	NR	University of Florida
Shirota et al. (44)	Randomized, participant- and outcome assessor blinded, controlled trial	(1) LF rTMS (2) HF rTMS	Sham	Nonmotor symptoms questionnaire (NMSQ) – Apathy subscore	All: 102 LF rTMS: 34 HF rTMS: 34 sham: 34	20 weeks	Japan	November 2008 to October 2010	NR
Wei et al. (36)	Randomized, participant-blinded, controlled trial	HF rTMS	Sham	Starkstein Apathy Scale (SAS)	NR	2 weeks	China	NR	Affiliated Hospital of Southwest Medical University
Primary progressive aphasia									
Pytel et al. (42)	Randomized, participant- and outcome assessor-blinded, cross-over trial	HF rTMS	Control-site stimulation	Neuropsychiatric Inventory-Apathy subscale (NPI-AS)	All: 27 active-site rTMS: 20 control-site stimulation: 7	16 weeks	Spain	2018 to 2020	Department of Neurology of Hospital Clinico San Carlos, Madrid
Mild cognitive impairment									
Cirillo et al. (35)*	Randomized, participant and outcome assessor blinded, controlled trial	HF rTMS	Sham	Apathy Evaluation Scale (AES)	All: 20 rTMS: 10 sham: 10	24 weeks	Italy	January 2018 to February 2020	First Division of Neurology of the University of Campania “Luigi Vanvitelli,” Naples, Italy
Esposito et al. (37)*	Randomized, participant- and outcome assessor-blinded, controlled trial	HF rTMS	Sham	Apathy Evaluation Scale (AES)	All: 23 rTMS: 11 sham: 12	4 weeks	Italy	January 2018 to February 2020	First Division of Neurology of the University of Campania “Luigi Vanvitelli,” Naples, Italy
Padala et al. (40)	Randomized, participant-, study personnel-, and outcome assessor-blinded, cross-over trial	HF rTMS	Sham	Apathy Evaluation Scale-Clinician version (AES-C)	All: 9	12 weeks	USA	NR	Department of Veterans Affairs Medical Center, USA
Chronic stroke									
Sasaki et al. (43)	Randomized, participant- and outcome assessor-blinded, controlled trial	HF rTMS	Sham	Apathy Scale (AS)	All: 13 rTMS: 7 sham: 6	5 days	Japan	NR	NR
Traumatic brain injury									
Meek et al. (45)	Single group before-after design study	HF rTMS	NA	Apathy Evaluation Scale (AES)	All: 15	3 weeks	Canada	NR	Neuropsychiatry and Neuromodulation Unit at Saint Boniface General Hospital

DLPFC, Dorsolateral prefrontal cortex; HF rTMS, High-frequency repetitive transcranial magnetic stimulation; LF rTMS, Low-frequency repetitive transcranial magnetic stimulation; M1, Primary motor cortex; NA, Not applicable; NR, Not reported; rTMS, repetitive transcranial magnetic stimulation; tDCS, transcranial direct current stimulation. *There is an overlap among the enrolled participants in Cirillo 2023 and Esposito 2022.

Overall, the meta-analysis of effectiveness data was not feasible due to the considerable heterogeneity observed in the clinical variables (e.g., differences in the populations and intervention used, inconsistent apathy outcomes measure used, and varying follow-up length) and methodological designs in the included studies.

Table 2 shows the summary of the population characteristics in the included studies. A total of 418 patients (overall female-to-male ratio 1:1.17) were included. It is worth noting that there was an overlap in the participants enrolled in the studies conducted by Cirillo in 2023 and Esposito in 2022. There were 4 studies each that focused on patients with Alzheimer’s (AD) (*n* = 143) (38, 41, 46, 47) and Parkinson’s disease (PD) (34, 36, 39, 44) (*n* = 187), 3 studies involving patients with MCI (*n* = 56) (35, 37, 40); and 1 study each for patients with chronic stroke (*n* = 13) (43), mild TBI (*n* = 15) (45), and primary progressive aphasia (PPA) (*n* = 20) (42). The majority of the studies were conducted in Europe (France (46, 47), Italy (35, 37), and Spain (42)) and North America (Canada (34, 45), United States (34, 39–41)), and 4 studies were performed in East Asia [China (36, 38) and Japan (43, 44)].

**Table 2:** Characteristics of the population in the included studies.

Study name	Age years	Sex Female, *n* (%)	Social construct of race/Ethnicity, *n* (%)	Education years	Comorbidiites *n* (%)	Concomittant medications *n* (%)
Alzheimer’s disease						
Hu et al. (38)	Mean (SD) rTMS + tDCS: 79.3 (6.2) rTMS: 76.9 (6.1) tDCS: 77.1 (6.9) Sham: 75.3 (5.7)	rTMS + tDCS: 9 (42.9) rTMS: 13 (61.9) tDCS: 13 (61.9) Sham: 11 (52.4)	NR	Mean (SD) rTMS + tDCS: 12.4 (4.1) rTMS: 13.0 (3.7) tDCS: 10.9 (4.6) Sham: 11.2 (4.4)	NR	Memantine: 67 (79.8) ChEIs: 34 (40.5)
Moreno et al. (47)	Mean (range) 73 (61–83)	5 (25)	NR	NR	NR	SSRI: 6 SNRI: 4 Tetracyclics: 2 BZN: 3 AAP: 4
Nguyen et al. (46)	Mean (SEM) 73.0 (7.2)	5 (50)	NR	NR	NR	NR
Padala et al. (41)	Mean (SD) rTMS: 74.3 (5.7) Sham: 79.6 (7.7)	rTMS: 1 (11) Sham: 1 (9)	Non-Hispanic Caucasian: 16 (80) Non-Hispanic African-American: 2 (10) Hispanic: 2 (10)	n (%) Less than High School: 1 (5) High School diploma: 9 (45) Some college degree: 4 (20) Bachelor’s degree: 4 (20) Professional/Graduate degree: 2 (10)	HPN: 13 (65) DM: 9 (45) Depression: 3 (15) CAD: 6 (30) Hypothyroidism: 0 Hyperlipidemia: 11 (55) DJD: 1 (5) Hearing Loss: 1 (5)	ADM: 9 (45) ChEI: 8 (40) Memantine: 2 (10)
Parkinson’s disease						
Brys et al. (34)	Mean (SD) M1 rTMS and DLPFC rTMS: 64.9 (8.0) M1 rTMS and DLPFC sham: 59.6 (12.6) M1 sham and DLPFC rTMS: 64.6 (12.3) M1 sham and DLPFC sham: 64.0 (7.4)	M1 rTMS and DLPFC rTMS: 9 (45) M1 rTMS and DLPFC sham: 5 (35.7) M1 sham and DLPFC rTMS: 6 (50) M1 sham and DLPFC sham: 4 (26.7)	NR	NR	NR	Patients taking ≥ 1 ADM: M1 rTMS and DLPFC rTMS: 12 (60) M1 rTMS and DLPFC sham: 10 (71.4) M1 sham and DLPFC rTMS: 8 (66.7) M1 sham and DLPFC sham: 9 (60)
NCT00955032	Mean (SD) rTMS: 63.8 (7.2) Sham: 72.8 (5.7)	rTMS: 2 (11) Sham: 3 (27.3)	NR	NR	NR	NR
Shirota et al. (44)	Mean (SD) LF rTMS: 68.8 (7.6) HF rTMS: 67.9 (8.4) Sham: 65.7 (8.5)	LF rTMS: 22 (61.1) HF rTMS: 22 (64.7) Sham: 17 (47.2)	NR	NR	NR	Antiparkinsonian drugs
Wei et al. (36)	rTMS: NR Sham: NR	rTMS: NR Sham: NR	NR	NR	NR	Antiparkinsonian drugs
Primary progressive aphasia						
Pytel et al. (42)	Mean (SD) Active-site rTMS: 66.9 (7.2) Control-site: rTMS: 66.1 (7.3)	Active-site rTMS: 12 (60) Control-site rTMS: 4 (67.1)	NR	Mean (SD) Active-site rTMS: 13.4 (4.4) Control-site rTMS: 13.9 (3.2)	NR	NR
Mild cognitive impairment						
Cirillo et al. (35)	Median (IQR) rTMS: 66.50 (62.2, 74,2); Sham: 70.50 (62.0, 75.0)	rTMS: 6 (60) Sham: 6 (60)	NR	Median (IQR) rTMS: 13.00 (9.5, 13.0) Sham: 10.50 (8.0, 13.0)	NR	NR
Esposito et al. (37)	Median (IQR) rTMS: 64 (60, 74) Sham: 70.50 (62.5, 77.2)	rTMS: 5 (45.4) Sham: 8 (50)	NR	Median (IQR), in years MCI-TMS: 13 (10, 13) MCI-C: 11 (8, 13)	NR	NR
Padala et al (41)	Mean (SD) rTMS-sham: 68.0 (10.0) Sham-rTMS: 64.0 (9.0)	rTMS-sham: 0 Sham-rTMS: 1 (20)	Non-Hispanic Caucasian: 4 (44) Non-Hispanic African-American: 5 (56) rTMS-Sham: Non-Hispanic Caucasian: 2 (50) Non-Hispanic African-American: 2 (50) Sham-rTMS: Non-Hispanic Caucasian: 2 (40) Non-Hispanic African-American: 3 (60)	n (%) High school diploma: 7 (78) Bachelor’s degree: 1 (11) Professional/graduate degree: 1 (11)	HPN: 5 (56) DM: 3 (33) Depression: 5 (56) CAD: 2 (22) Hypothyroidism: 2 (22) Hyperlipidemia: 5 (56) DJD: 5 (56) Hearing Loss: 5 (56) MCI: 9 (100)	ADM: 4 (44) ChEI: 0 (0) Memantine: 0 (0)
Chronic stroke						
Sasaki et al. (43)	Mean (SD) rTMS: 66.1 (11.2) Sham: 62.8 (10.1)	rTMS: 2 (29) Sham: 0	NR	NR	NR	NR
Traumatic brain injury						
Meek et al. (45)	Mean (SD) 46.1 (15.8)	8 (53.3)	NR	Mean (SD) 28.8 (1.3)	NR	NR

AAP, Atypical antipsychotics; ADM, Antidepressant medications; BZN, Benzodiazepines; CAD, Coronary artery disease; ChEI, Anticholinesterase inhibitors; DLPFC, Dorsolateral prefrontal cortex; DJD, Degenerative joint disease; DM, Diabetes mellitus; HF, High-frequency repetitive transcranial magnetic stimulation; IQR, Interquartile range; LF rTMS, Low-frequency repetitive transcranial magnetic stimulation; MCI, Mild cognitive impairment; rTMS, Repetitive transcranial magnetic stimulation; HPN, Hypertension; M1, Primary motor cortex; NR, Not reported; SEM, Standard error of the mean; SD, Standard deviation; tDCS, Trancranial direct current stimulation.

### 3.2. Interventions employed in the included studies

Nearly all of the included studies employed high-frequency (HF) rTMS as an intervention (34–44, 46, 47), whereas 2 studies used low-frequency (LF) rTMS (44, 45). Two studies used a combination of HF rTMS and cognitive training (“NeuroAD”) (46, 47), while one study utilized a combination of HF rTMS and transcranial Direct Current Stimulation (tDCS) (38). The median intensity of stimulation was 100% of the resting motor threshold (RMT) (ranging from 80 to 120% RMT). Among the clinical trials included, the majority of the studies utilized various versions of sham stimulation in the comparison group (34–36, 38–41, 43, 44); 1 study used tDCS as an active stimulation in the control group (38); 1 study utilized a control-site stimulation in the comparison group (42). In terms of stimulation sites, 2 studies targeted the prefrontal area (39, 43), 3 studies stimulated the left dorsolateral prefrontal cortex (DLPFC) (34, 40, 41), 2 studies targeted the bilateral DLPFC (35, 37), 2 studies targeted the right DLPFC with high-frequency (36) and low-frequency stimulation (45), 1 study targeted the bilateral angular gyrus (38), 1 study stimulated the supplementary motor area (44), and 3 studies had complex stimulation sites (42, 47). Other rTMS parameters, including session duration and treatment course, varied extensively across the studies (see Table 3).

**Table 3:** Characteristics of the interventions in the included studies.

Study name	rTMS protocol	Stimulus intensity % RMT	Frequency Hz	Coil type	Stimulation site	Stimulation site navigation	Session duration	Treatment course	Pulses per day	rTMS device
Alzheimer’s disease										
Hu et al. (38)	HF rTMS+ tDCS	90	40	Figure-of-8 coil	Bilateral angular gyrus	Scalp-based navigation with EEG guidance	30 min	Treatment given on alternate days, three times a week for 4 weeks	1200 stimuli for each unilateral angular gyrus	Electromagnetic Stimulator® (Tianjin, China)
Moreno et al. (47)	NeuroAD (HF rTMS)	NR	10	NR	Multiple brain sites	NR	1 h	5 daily sessions per week with 3 brain areas stimulated per session over 6 weeks	NR	NR
Nguyen et al. (46)	NeuroAD (HF rTMS)	100	10	Figure-of-8 coil	Multiple brain sites	MRI guidance navigation	30 min	1 session per day, 5 days a week for 5 weeks	400	Neuronix®
Padala et al. (40)	HF rTMS	120	10	XPLOR treatment coil	Left DLPFC	Scalp-based navigation	NR	5 days a week for 4 weeks	3,000	NeuroStar® TMS Therapy and XPLOR System
Parkinson’s disease										
Brys et al. (34)	HF rTMS	NR	10	Figure-of-8 coil	Left DLPFC and bilateral M1 (primary motor cortex)	Scalp-based navigation	50 min	5 daily sessions per week over 2 weeks	2,000 stimuli for the left DLPFC and 1,000 stimuli for each M1	Magstim Super-Rapid stimulator® (Wales, UK)
NCT00955032	HF rTMS	80 and 90	5	NR	Left prefrontal area	NR	25 min	10 days of consecutive sessions	2,000	NR
Shirota et al. (44)	(1) LF rTMS (2) HF rTMS	110	(1) 1 Hz (2) 10 Hz	Figure-of-8 coil	Supplementary motor area	Scalp-based navigation	(1) LF rTMS: 17 min (2) HF rTMS: 20 min	Weekly intervention for 8 weeks	1,000	Magstim Rapid® (UK)
Wei et al. (36)	HF rTMS	110	10	Figure-of-8 coil	Right DLPFC	Neuroimaging -guided navigation	NR	10 sessions for 5 days per week for a total of 20 sessions over 2 weeks.	1,500	YINGCHI® (Shenzhen, China)
Primary progressive aphasia										
Pytel et al. (42)	HF rTMS	100	20	Figure-of-8 coil	*Target personalization	*Target personalization	NR	15 sessions on consecutive working days	1,500	Magstim Rapid2 stimulator®
Mild cognitive impairment										
Cirillo et al. (35)	HF rTMS	80	10	Figure-of-8 coil	Bilateral DLPFC	Scalp-based navigation	10 min	5 days per week over 4 weeks	2,000	Magstim2 Rapid stimulator®
Esposito et al. (37)	HF rTMS	80	10	Figure-of-8 coil	Bilateral DLPFC	Scalp-based navigation	10 min	5 days per week over 4 weeks	2,000	Magstim2 Rapid stimulator (Whitland, UK)
Padala et al. (41)	HF rTMS	120	10	XPLOR treatment coil	Left DLPFC	Scalp-based navigation	NR	5 days per week over 2 weeks	3,000	NeuroStar® TMS Therapy and XPLOR System
Chronic stroke										
Sasaki et al. (43)	HF rTMS	80	10	Double-cone coil	Dorsal anterior cingulate cortex (dACC) to medial prefrontal cortex (mPFC)	MRI guidance navigation	20 min	5 sessions over 5 days	2,000	Mag-Pro R30 stimulator (Farum, Denmark)
Traumatic brain injury										
Meek et al. (45)	LF rTMS	110	1	Figure-of-8 coil	Right DLPFC	MRI guidance navigation	NR	2 sessions administered each day for a total of 30 sessions over 3 weeks	1,200	Magstim Rapid2 rTMS system

DLPFC, Dorsolateral prefrontal cortex; EEG, Electroencephalogram; HF rTMS, High-frequency repetitive transcranial magnetic stimulation LF rTMS, Low-frequency repetitive transcranial magnetic stimulation; MRI, Magnetic resonance imaging; NR, Not reported; RMT, Resting motor threshold; rTMS, Repetitive transcranial magnetic stimulation; tDCS, Transcranial direct current stimulation. *Target personalization: The participants were administered with excitatory and/or inhibitory protocols at a varying number of brain sites, specifically between 6 and 10, depending on their PPA phenotype and neuroimaging findings.

### 3.3. Outcome measures used in the included studies

There was a considerable degree of variation in the outcome measures that were used to evaluate apathy in the included studies (see Table 1). Seven studies employed the Apathy Evaluation Scale (AES) (34, 35, 37, 39–41, 45) and 2 studies used the Neuropsychiatric Inventory–Apathy subscale (NPI-AS) (38, 42); and 2 utilized the Apathy Inventory (AI) (46, 47). Other apathy tools utilized were as follows: Nonmotor symptoms questionnaire–Apathy Subscore (NMSQ-AS) (44); 1 study used the Starkstein Apathy Scale (SAS) (36); 1 study used the Lille Apathy Rating Scale (LARS) (39); and 1 study used the Apathy Scale (AS) (43). Five studies specifically focused on apathy as the primary outcome (36, 40, 41, 43, 47).

### 3.4. Risk of bias in the included studies

**Figure 2:**
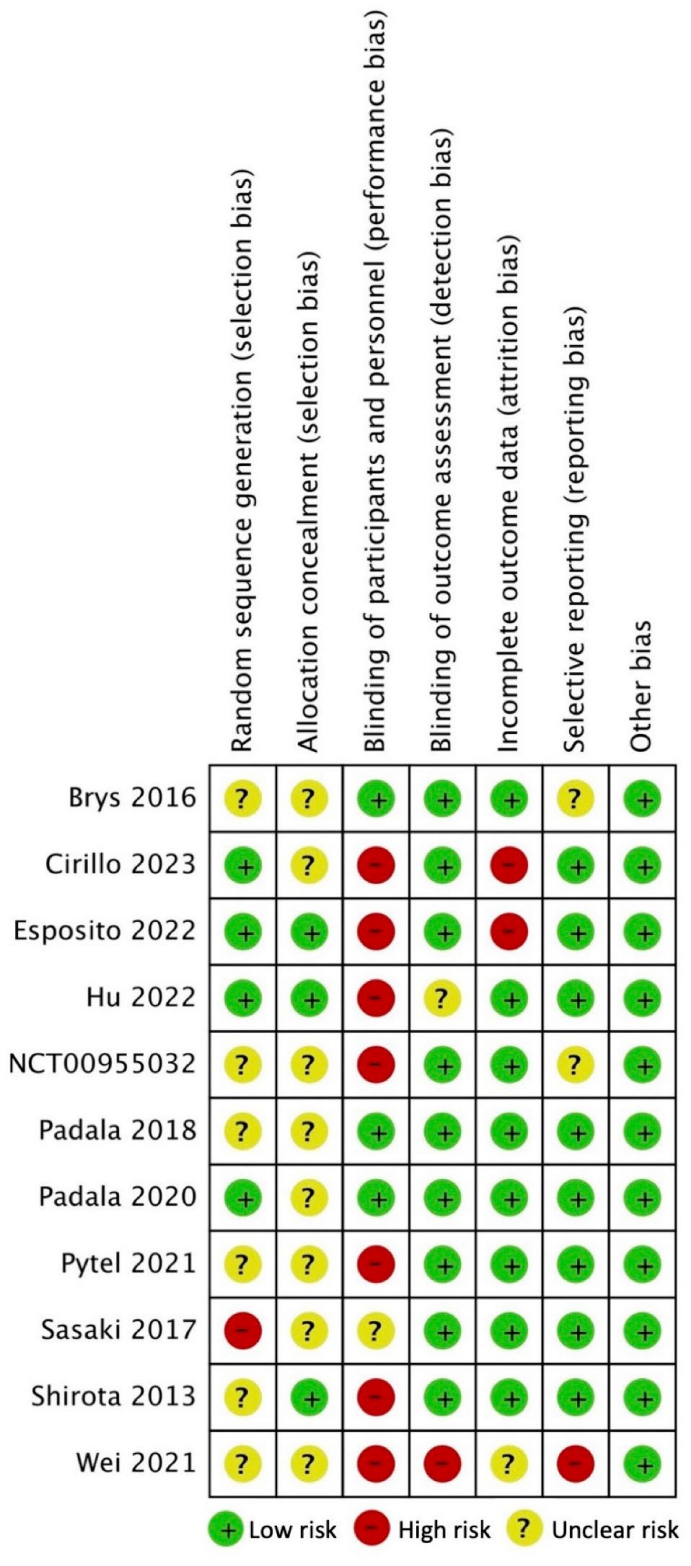
Risk of bias summary for included clinical trials: review authors’ judgments about each risk of bias item for each included study.

**Figure 3:**
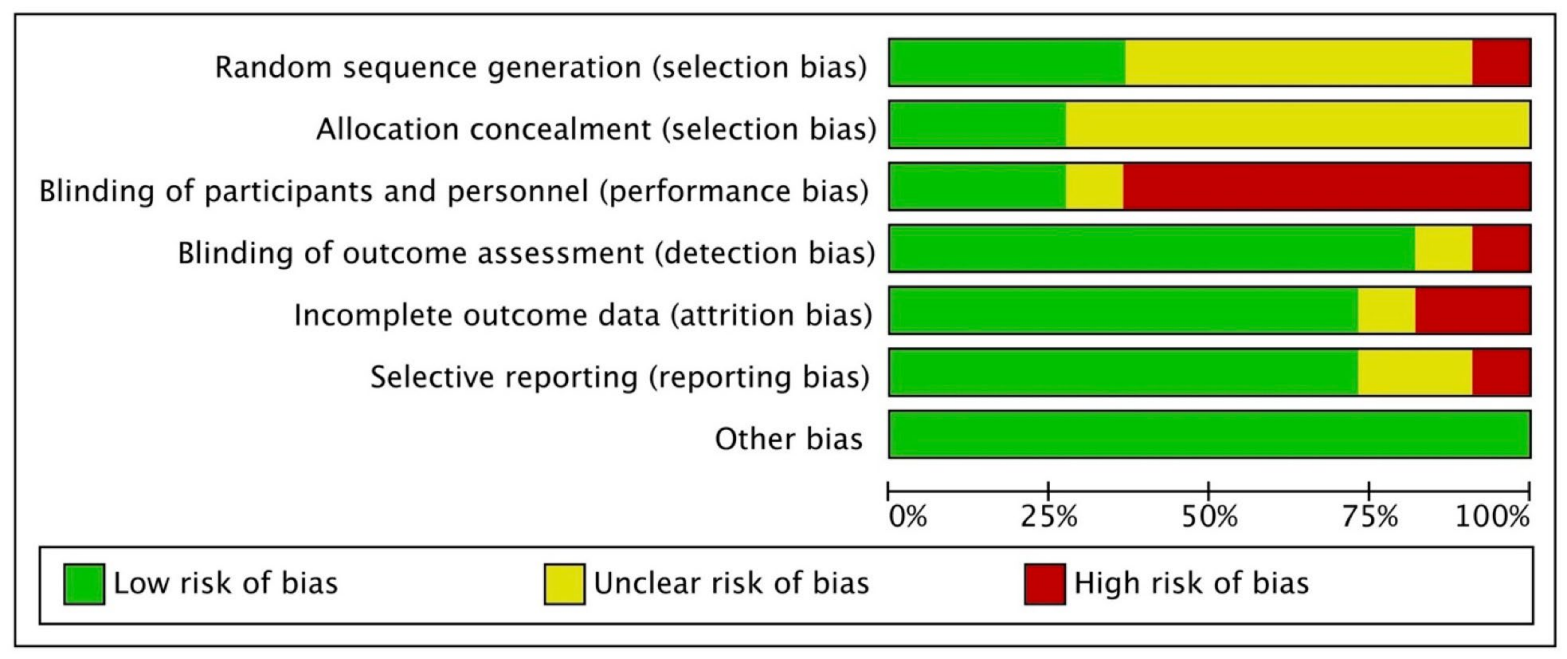
Review authors’ judgments about each risk of bias item presented as percentage across all included studies.

A considerable proportion of the studies exhibited an unclear risk with regards to selection bias, primarily stemming from inadequate disclosure of the randomization process and allocation concealment (34–36, 39–44). In the context of generating random sequences, it was observed that only four studies were classified as having a low risk (35, 37, 38, 41), whereas 1 study was identified as having a high risk of bias (43). Regarding allocation concealment, only 3 studies were deemed to have a low risk of bias (37, 38, 44). Seven studies were classified as high risk for performance bias because only the participants were blinded and not the study personnel/rTMS technicians (35–39, 42, 44); 3 studies were considered low risk for this domain (34, 40, 41). Nine studies implemented the blinding of assessors (34, 35, 37, 39–44) whereas 1 study did not (36). Regarding bias due to incomplete outcome data, 8 studies had low attrition rate (34, 38–40, 42–44, 49) while one study did not specify the flow of participants (36). Two studies displayed unclear risk for reporting bias due to inadequate reporting of the results of statistical analyzes conducted for the apathy outcome (34, 39); 1 study was deemed high risk (36) while the rest were considered low risk (35, 37, 38, 40–44) for selective reporting of results. The included 11 RCTs studies appeared to be free from other sources of bias (34–44). Overall, the included RCTS exhibited a level of methodological quality ranging from fair to good. Figures 2, 3 display the summary RoB assessments. In addition, the methodological quality of the 3 studies with a single-group, before-after design was considered fair (see Table 4).

**Table 4:** Methodological quality assessment for included studies with a single group, before-after designs.

Risk of bias assessment questions	Meek 2020	Moreno 2022	Nguyen 2017
1. Was the study question or objective clearly stated?	Y	Y	Y
2. Were eligibility/selection criteria for the study population prespecified and clearly described?	Y	Y	CD
3. Were the participants in the study representative of those who would be eligible for the test/service/intervention in the general or clinical population of interest?	N	Y	Y
4. Were all eligible participants that met the prespecified entry criteria enrolled?	CD	Y	CD
5. Was the sample size sufficiently large to provide confidence in the findings?	N	CD	N
6. Was the test/service/intervention clearly described and delivered consistently across the study population?	Y	Y	Y
7. Were the outcome measures prespecified, clearly defined, valid, reliable, and assessed consistently across all study participants?	Y	N	Y
8. Were the people assessing the outcomes blinded to the participants’ exposures/interventions?	N	N	N
9. Was the loss to follow-up after baseline 20% or less? Were those lost to follow-up accounted for in the analysis?	Y	CD	CD
10. Did the statistical methods examine changes in outcome measures from before to after the intervention? Were statistical tests done that provided *p* values for the pre-to-post changes?	Y	Y	Y
11. Were outcome measures of interest taken multiple times before the intervention and multiple times after the intervention (i.e., did they use an interrupted time-series design)?	N	Y	N
12. If the intervention was conducted at a group level (e.g., a whole hospital, a community, etc.) did the statistical analysis take into account the use of individual-level data to determine effects at the group level?	NA	NA	NA
Quality rating	Fair	Fair	Fair
CD, Cannot determine; N, No; NA, Not applicable; Y, Yes.			

### 3.5. Effects of the rTMS interventions on apathy

#### 3.5.1. Alzheimer’s disease

In a randomized, participant-, rTMS personnel-, and outcome assessor-blinded study by Padala et al. (41) (*n* = 20; trial registration: NCT02190084), the effects of HF rTMS (*n* = 9) at left DLPFC administered for 20 sessions over 4 weeks was compared to sham stimulation (*n* = 11) in terms of the AES-clinician version (AES-C) among patients with AD diagnosed using the Diagnostic and Statistical Mental Disorders-Text Revision (DSM-IV-TR) criteria. Pertinent baseline clinical variables including age, sex, education profile, concomitant medications and comorbidities, as well as pretreatment baseline measures including AES-C were similar in both groups. Following a period of 4 weeks of post-intervention monitoring, a significant difference was observed between rTMS [mean (95% CI), −11.0 (−15.2 to −6.7)] and sham groups [−0.8 (−4.8 to 3.2)] with regard to the AES-C score change [−10.1 (−15.9 to −4.3); *p* = 0.002] using analysis of covariance (ANCOVA). After 4 weeks, within-group analyzes indicated a statistically significant improvement in AES-C following rTMS treatment, with an average reduction of 11.0 points from the initial measurement (*p* < 0.001) while within-group sham treatment did not lead to a significant change in AES-C (*p* = 0.662). However, after 8 weeks and 12 weeks, the effects were not statistically durable in the within-group rTMS analyzes using repeated measures mixed model [−3.5 (−9.6 to 2.6)] and [ − 4.4 (−10.6 to 1.8), respectively].

Hu et al. (38) conducted a randomized controlled trial involving probable AD patients (reported to be diagnosed using the National Institute on Aging-Alzheimer’s Association (NIA-AA) guidelines on neuropathological assessment of AD) that assessed apathy using the NPI-AS with 4 treatment arms each with a sample size of 21 (1): HF rTMS + tDCS (2); HF rTMS (3); tDCS only; and (4) sham stimulation. The blinding procedures were not adequately described. Age, sex, years of education, AD duration, co-medications, and other measures were not considerably different among the 4 arms. The stimulated sites were the bilateral angular gyrus and the treatment course was given on alternate days, 3 times per week, over a period of 4 consecutive weeks. Pairwise comparisons showed that the combined regimen of HF rTMS+tDCS yielded significantly greater improvement than sham in NPI-AS from baseline and at 4 (*p* < 0.001) and at 12 weeks (*p* = 0.002); however, no significantly different results were found between rTMS + tDCS regimen and either rTMS-only or tDCS-only (both *p* > 0.017). Significant improvements in NPI-AS compared to baseline were observed within the rTMS+tDCS group after 4 (*p* < 0.001) and after 12 weeks (*p* = 0.001).

Moreno et al. performed a retrospective, pre−/posttest designed study (*n* = 30) without a control group to test the impact of NeuroAD procedure (HF rTMS + cognitive training) on AI scores in patients with dementia due to probable AD followed up to 4 years (47). The treatment protocol consists of 5 sessions per week, conducted over a period of 6 weeks, during which 3 specific brain areas are stimulated in each session. The relevant description of NeuroAD procedure was further detailed elsewhere (50, 51). While controlling for age, gender, disease duration, AD treatment, and psychiatric medication, linear mixed model analysis showed that NeuroAD improved AI scores at various time points compared to baseline: 6 weeks [*β* coefficient (95% CI), −8.7 (−11.1 to −6.3)], 3 months [−7.2 (−9.7 to −4.7)], 12 months [−6.8 (−9.5 to −4.1)], at a mean of 28 months [−5.5 (−8.5 to −2.5)] (all *p* < 0.001).

Nguyen et al. administered the NeuroAD regimen in AD patients (*n* = 10) followed over 6 months in a prospective study with a single-group, pre−/posttest design to test its effect on AI scores (46). The treatment course consisted of daily sessions for 5 days over 5 weeks. Repeated measures analysis of variance (ANOVA) showed that NeuroAD treatment significantly improved AI scores at 45 days (mean ± SE, 17.4 ± 2.7) and at 6 months (9.4 ± 1.8) compared to baseline (17.4 ± 2.2; *p* = 0.0125).

#### 3.5.2. Parkinson’s disease

Brys et al. conducted a randomized, participant-, study personnel-, and outcome assessor-blinded, controlled trial with an aim to determine the effects of HF rTMS on AES compared to sham on patients with PD (*n* = 61) followed over 6 months (34). The rTMS interventions targeted the following sites (1): both the bilateral primary motor cortex (M1) + left DLPFC (*n* = 20) (2); M1 + sham (*n* = 14) (3); left DLPFC + sham (*n* = 12) (4); double sham (*n* = 15); the treatment course consists of 5 sessions per week over 2 weeks. Baseline characteristics such as age, sex, disease duration, Unified Parkinson’s Disease Rating Scale (UPDRS) scores, among others, appear to be similar across the groups. Using repeated-measures ANOVA, the results showed no significant difference among the treatment groups in AES scores at 1 week, 1 month, 3 months and 6 months after treatment compared to baseline (comparative statistical results unreported). The authors also concluded that there was no evidence of a synergistic effect of stimulating both the M1 and DLPFC (statistical results unreported).

In NCT00955032 (“ReStore Study”), the investigators tested the impact of HF rTMS (*n* = 16) vs. sham (*n* = 8) on AES and LARS scores among PD patients (*n* = 24) with a follow-up period of 10 days via implementing a randomized, participant- and outcome assessor-blinded, controlled trial (39, 48). Age and sex attributes appear to be comparable in both groups. The stimulation was applied to the left prefrontal area and was administered daily over 10 days. However, there were no significant differences between rTMS and sham in the change from baseline and 10-day posttreatment AES (rTMS, in mean ± SD: 19.6 ± 5.1 (baseline) to 17.9 ± 7.0 (posttreatment); sham: 18.8 ± 3.9 to 16.8 ± 4.6) or in LARS (rTMS: −15.6 ± 9.1 to −20 ± 7.8; sham: −18.9 ± 4.5 to −22.1 ± 5.2; comparative statistical methods/results unreported).

Shirota et al. (*n* = 102) performed a randomized, participant- and outcome assessor-blinded, controlled trial comparing LF rTMS (*n* = 34), HF rTMS (*n* = 34) and sham (*n* = 34) arms on their effects on improving the NMSQ-AS scores in the PD population followed over 20 weeks (44). Pretreatment characteristics, including current age, age at onset, sex, Hoehn-Yahr (H&Y) stage, disease duration, and current levodopa equivalent dose were considered similar in the 3 arms. The stimulation site was set at 3 cm anterior to the leg motor area along with the midline targeting the supplementary motor area and the protocol consists of weekly administration of treatment over 8 weeks (52, 53). Using ANOVA, results indicate that LF rTMS, HF rTMS and sham groups’ mean (SD) change in NMSQ-AS scores from baseline and at week 9 (0.3 ± 1.0, 0.4 ± 1.2, 0.7 ± 1.3, respectively; *p* = 0.55) and at week 20 (0.0 ± 1.0, 0.1 ± 1.4, 0.2 ± 1.0, respectively; *p* = 0.83) did not show statistically significant differences.

Wei et al. conducted a randomized, participant-blinded, controlled trial with an aim to test HF rTMS’ impact on SAS scores in PD patients with a follow-up period of 2 weeks (36). The study primarily compared apathetic and non-apathetic PD patients classified using Marin’s criteria (54); however, the number of individuals who received rTMS or sham, as well as their baseline characteristics, is unclear from the published paper. The stimulation protocol comprises 10 sessions per week, for a total of 20 sessions over the course of 2 weeks. Among those classified with apathy, results demonstrate that there was a significant difference between rTMS vs. sham in terms of improvement in SAS scores after 2 weeks (*p* = 0.005) using the Wilcoxon test. The findings of the study also suggest that there was a significant difference in the posttreatment SAS scores between the apathetic group and the non-apathetic group (*p* < 0.0001); however, this difference can be attributed to the substantial difference in their baseline SAS scores as well (*p* < 0.0001).

#### 3.5.3. Primary progressive aphasia

Pytel et al. (*n* = 27) undertook a randomized, participant- and outcome-assessor-blinded, cross-over trial study with an objective to determine the effect of active-site HF rTMS (*n* = 20) vs. control-site (*n* = 7) stimulation on NPI-AS scores in patients with PPA followed over 16 weeks (trial registration: NCT03580954) (42, 55). Baseline clinical parameters, including age, sex, years of education, PPA clinical phenotype and symptom duration, in both groups were comparable. The patients were exposed to excitatory and/or inhibitory protocols in a range of 6 to 10 distinct brain sites, based on PPA phenotype and neuroimaging results (i.e., *“target personalization”*). The control-site stimulation was administered over the vertex, which was described elsewhere (56). The stimulation protocol involved 15 sessions on consecutive working days. The active-site intervention group showed significant improvements in NPI-AS scores from baseline to post-intervention compared to the control group (mean difference ± SD, −1.9 ± 2.8 vs. 0.0 ± 3.0, respectively; *p* = 0.03) analyzed using the Mann–Whitney U test.

#### 3.5.4. Mild cognitive impairment

Both Esposito et al. (*n* = 21) and Cirillo et al. (*n* = 20) conducted controlled trials on the impact of HF rTMS on AES compared to sham among the MCI population (reported to be diagnosed using the NIA-AA workgroup guidelines on MCI diagnosis due to AD), with randomization, and blinding of participants and outcome assessors (35, 37). Given the similarities between the two trials, it is highly probable that they included overlapping cohorts of patients with MCI; however, these trials differ in the report of follow-up length. The treatment course in these studies involved stimulation of the bilateral DLPFC administered for 20 sessions over 4 weeks. Age, sex, and years of education were similar between the rTMS and sham in both studies. In Esposito et al., AES did not significantly differ among rTMS {median [interquartile range (IQR)], 37.0 [35.0 to 39.0], *n* = 11}, sham [35.0 (29.5 to 39.5), *n* = 12] and healthy controls [28.0 (24.0 to 31.5), *n* = 12] at 4 weeks of intervention (adjusted *p* = 0.213). A similar conclusion was also observed in Cirillo 2023, where the rTMS [median (IQR), 36.5 (34.8 to 41.8), *n* = 10] and sham [34.5 (28.0 to 40.2), *n* = 10] groups did not vary substantially in terms of AES scores at 4 weeks (adjusted *p* = 0.983). Furthermore, Cirillo et al. (35, 37)did not find a significant difference between rTMS [median (IQR), 33.0 (27.0 to 46.5)] and sham [37.0 (31.0, 46.5)] at 6 months (adjusted *p* = 0.586). All analyzes were conducted using ANCOVA utilizing pretreatment score as a covariate.

Padala et al. (*n* = 9) performed a randomized, participant-, study personnel/rTMS technician-, and outcome assessor-blinded, cross-over trial examining the effect of left DLPFC HF-rTMS compared to sham on AES-C among the MCI cohort diagnosed using the Petersen’s criteria (trial registration: NCT02190019) (40, 57). The treatment protocol entailed undergoing treatment for a duration of 5 days within a span of 2 weeks, after which a treatment-free interval of 4 weeks was observed. Participants subsequently underwent a cross-over design, wherein they received the alternative treatment for a duration of 2 weeks. A final evaluation was conducted during the 12-week follow-up appointment. The baseline characteristics of the two groups (i.e., rTMS-sham and sham-rTMS) were noted to be comparable in terms of age, sex, years of education, concurrent medications, and presence of comorbidities. Using mixed model ANCOVA and adjusted for pretreatment measure, crossover sequence, and week, a significant difference was observed in the change of AES-C scores between the active-coil [mean (95% CI), −7.4 (−11.9 to −2.8)] and the sham-coil treatment groups [−1.5 (−6.1 to 3.1); mean difference (95% CI), −5.9 (−11.6 to −0.2), *p* = 0.045] in MCI patients.

#### 3.5.5. Chronic stroke

Sasaki et al. undertook a randomized, participant- and outcome assessor-blinded controlled study to examine the effects of HF rTMS on AS scores compared to sham among chronic stroke patients who were followed over 5 days. The treatment regimen consisted of 5 sessions administered consecutively over a span of 5 days. The targeted area for stimulation was the upper-middle region of the forehead, extending from the external auditory meatus to a point 30° above the orbitomeatal line (i.e., dorsal anterior cingulate cortex (dACC) to medial prefrontal cortex (mPFC)). Age, sex, stroke subtype, and laterality of the brain lesion were all comparable between the rTMS and sham groups. The number of years between stroke onset and initiation of treatment was also comparable in both the rTMS group (mean ± SD, 4.1 ± 2.9 years) and the sham group (5.3 ± 5.7 years). The findings of the study indicate that there was a statistically significant difference in the extent of change observed in the AS score between the group that received rTMS (47.5% ± 31.9) and the group that received sham stimulation (1.7% ± 27.8; *p* = 0.02) in chronic stroke patients analyzed using the Mann–Whitney U test. The within-group rTMS arm demonstrated a significant improvement from pre- to posttreatment AS scores (mean ± SD, 15.9 ± 6.3 vs. 9.3 ± 6.0, respectively; *p* = 0.02), whereas the sham stimulation group nonsignificant pre−/posttreatment changes in the AS score (14.3 ± 7.4 vs. 13.8 ± 8.3, respectively, *p* unreported) evaluated using the Wilcoxon signed-rank test.

#### 3.5.6. Traumatic brain injury

Meek et al. (*n* = 15) conducted a pilot study with pre-post design (no control group) with an aim to examine the effect of LF rTMS on right DLPFC to improve AES scores in patients with mild TBI followed over 3 weeks (45). The mean (SD) months post-injury was 20.4 ± 14.6. The rTMS procedure comprised 2 sessions administered each day totaling 30 sessions over 3 weeks. The paired t-test results revealed no significant difference in mean (SD) baseline and posttreatment scores (50.5 ± 10.3 vs. 50.6 ± 11.4, respectively; *p* = 0.334) after 3 weeks.

### 3.6. Safety profile

The studies included in this review did not report any serious AEs related to the rTMS protocols. The majority of studies have reported occasional instances of discomfort/pain/shock sensations at the stimulation sites, including headaches. In contrast, few studies have documented rare occurrences of fatigue, scalp burn, scalp numbness, nausea, dizziness, lightheadedness, head pressure, twitching of the eyes/face, shock sensations in the eyes, perception of phantom smells, toothache, insomnia, tinnitus, back pain, and pitting edema. The AEs observed were generally mild, transient and did not necessitate symptomatic use of medications.

## 4. Discussion

Our review provides a comprehensive assessment of the potential efficacy and tolerability of rTMS protocols in modifying apathy among individuals diagnosed with neurodegenerative conditions (including AD, PD, and PPA), MCI, chronic stroke, and traumatic brain injury. In summary, our review reveals the following insights (1): all included AD trials indicate beneficial signal on apathy for those exposed to rTMS (2); the prevailing body of studies on PD has yielded non-significant results (3); a single study (RCT) involving patients with PPA demonstrated significant positive effects (4); the MCI trials yielded varying conclusions (5); one study (RCT) on chronic stroke found rTMS may improve apathy (6); one study on mild TBI found no significantly favorable association; and (7) the implementation of various rTMS protocols on the populations described is generally safe.

The findings from the 2 included randomized, sham-controlled trials suggested that rTMS may be beneficial in terms of improving apathy among AD (38, 41). These results were also supported by the 2 studies with single-group before-after designs utilizing a combination of rTMS plus cognitive training (46, 47). The durability of the effects remains uncertain, as Padala et al. observed positive effects restricted at the 4-week time mark, whereas Hu et al. (38) indicated beneficial effects not only at 4 weeks but also at 12 weeks when compared to baseline. Furthermore, both Nguyen et al. and Moreno et al. posited that the observed rTMS’ effects on apathy could potentially persist for a duration exceeding 6 months (46, 47). However, it is important to note that these investigations lacked a control group, thereby constraining the validity of their findings. Hu et al. (38) utilized a combination of rTMS and tDCS as the main intervention of interest which was found to be superior than sham in addressing apathy; however, when this mixed NIBS regimen was compared to rTMS-only or tDCS-only, no significant difference was found in their NPI-AS scores, suggesting that either NIBS regimen can potentially improve apathy and the efficacy of the combined NIBS regimen in alleviating apathy does not appear to surpass that of a single NIBS regimen.

On the other hand, inconsistencies were observed in the conclusions of the included studies regarding the MCI population. Results from the two published articles that describe an overlapping MCI cohort (35, 37), an investigation that targeted the bilateral DLPFC and used a treatment course of 20 sessions total over 4 weeks, showed non-significant difference in apathy scores at follow-up ranging from 4 weeks to 6 months in those with MCI. This is in contrast to the findings of Padala et al. that reported a statistical difference in apathy scores between rTMS and sham in a trial that targeted the left DLPFC and used a shorter course of 10 sessions over 2 weeks (41).

In the PD population, 4 randomized, sham-controlled trials were identified, which investigated the effects of rTMS on apathy (34, 36, 39, 44). Three studies comprised of Brys et al., NCT00955032, and Shirota et al. reported non-significant differences in apathy scores between rTMS and sham stimulation with follow-up duration ranging from 1 week to 6 months. Wei et al. (36) stands apart as the sole study that yielded results favoring rTMS over sham stimulation specifically in the context of PD individuals diagnosed with apathy using standard criteria, but this was noted only at a short course at 2 weeks posttreatment. Nevertheless, the absence of relevant information regarding the number of participants and descriptive statistics pertaining to the apathy scores in the study conducted by Wei et al. (36) gives rise to apprehensions owing to bias arising from the selective reporting of data.

In the RCT conducted by Pytel et al. on individuals diagnosed with PPA, the results indicated that theimplementation of active-site rTMS intervention yielded positive outcomes in terms of ameliorating apathy (42). In addition, Sasaki et al. trial reported that there was a significant improvement in apathy scores among those exposed to rTMS in patients with chronic stroke (43). However, Meek et al. revealed that rTMS was not associated with better apathy scores posttreatment in individuals with mild TBI (45).

Biological marker-based diagnosis for AD and MCI as reported by Jack et al. and Dubois et al. plays a crucial role in giving context to the observed effects of rTMS in the included AD and MCI studies (58–60). The inclusion criteria of Padala et al. involved a diagnosis of AD using the DSM-IV-TR criteria, an age threshold of ≥55, and Mini Mental State Examination (MMSE) score of ≥18. The limitation of the DSM-IV-TR criteria is that they do not specify the use of AD biomarkers as an indication that the dementia is caused by AD pathology. In Hu et al. (38), the authors incorporated these inclusion criteria of age 60 to 90 years, Clinical Dementia Rating (CDR) of 2, and MMSE of 10–20 and the article stated that the authors used the 2011 NIA-AA criteria for “probable AD” referencing the work by Montine et al. (61), a report that summarized the guidelines of the NIA-AA on the neuropathological assessment of AD by Hyman et al. (62). It is undefined in their reports whether an AD biomarker was used for their population, and it was not specifically indicated in their inclusion criteria. In Moreno et al., the age included in their population ranged from 61 to 83 years and it was mentioned that all of the patients included had “dementia due to AD” diagnosed in reference centers. Lastly, in Nguyen et al. (46), the authors stated that they included patients aged 61 to 84 with “probable AD” and with MRI findings consistent with AD diagnosis. The publications of Moreno et al. (47) and Nguyen et al. (46) did not specifically state whether or not the participants underwent an AD biomarker study and what AD diagnostic criteria were implemented.

In terms of the MCI diagnosis, it is also important to take into account how the studies diagnosed patients with MCI, as this condition represents a heterogeneous group that includes amnestic MCI (i.e., those at higher risk for developing AD dementia) and non-amnestic MCI (i.e., those at higher risk for developing non-AD dementias) (63). In Padala et al., the MCI criteria they utilized were based on Petersen’s criteria, and they set an MMSE score threshold of 23 or higher, and age cut-off of ≥55 years. Initially, Petersen’s criteria were centered on delineating the amnestic MCI cohort, but over time, they evolved to include the non-amnestic presentations. In the published article of Padala et al., the authors did not specify the MCI classifications of the enrolled participants in their trial. In Esposito et al. and Cirillo et al. (2023), the published articles indicated that they used the NIA-AA workgroup guidelines on the diagnosis of MCI due to AD, with a CDR of 0.5 and an age cut-off of ≥40 years. The MCI “core” clinical criteria of the 2011 NIA-AA workgroups cover impairments in one or more cognitive domains (64). The 2011 NIA-AA criteria also separately designated the term “MCI due to AD” to indicate evidence of an abnormal AD biomarker aside from fulfilling the core criteria (64). In the published article of Esposito 2022 and Cirillo 2023, although they specified that they used the core criteria, it is unclear whether they enrolled specifically those with “MCI due to AD,” and it is likely that they enrolled patients with MCI with impairments in various cognitive domains, who may be at risk for transitioning to non-AD dementias.

Overall, the inclusion criteria used in the diagnosis of AD dementia and MCI in the included studies did not mention the use of relevant AD biomarkers, which may diminish the internal validity of the results. It is crucial to acknowledge that distinct neurodegenerative disorders may have variable responses to rTMS due to heterogeneity in their pathological underpinnings.

The majority of the patients enrolled in the included AD studies had late-onset AD (LOAD) (i.e., ≥ 65 years). We theorize that the age of presentations in AD and MCI may also affect response to various rTMS parameters. Individuals with sporadic early-onset AD (EOAD) exhibit a more accelerated clinical deterioration and experience more pronounced deficits in attention, language, visuospatial abilities, and executive functions (65, 66). Furthermore, the typical presence of medial and lateral temporal and parietal atrophy may generally signal AD as the underlying cause; however, EOAD often has a greater tendency to affect the frontal lobe, while showing less involvement of the medial temporal lobe and a hippocampal sparing (65, 67–69). In the initial stages, those with EOAD tend to exhibit more diminished cortical metabolism and more atrophy than those with LOAD (65, 70, 71). While not statistically significant, EOAD patients had lower amyloid-beta 1–42 (Aβ1-42) and higher total Tau (t-Tau) levels in their cerebrospinal fluid (CSF), indicating a more rapid cognitive deterioration (72). Flurodeoxyglucose (FDG) positron emission tomography (PET) also demonstrated a substantial reduction in glucose intake in a large region of the left parietal lobe in EOAD patients as compared to LOAD (72). Although the pathologic substrate between EOAD and LOAD are similar, their clinical, CSF, and brain structural and metabolic differences provide evidence that rTMS protocols may demonstrate varying therapeutic effectiveness in several outcomes including the apathy domains.

PPA is under the spectrum of frontotemporal dementia (FTD), which encompasses several clinical presentations, including the behavioral variant FTD (bvFTD). bvFTD manifests with a remarkably high incidence of apathy, ranging from 62 to 89% (73, 74). In the study conducted by Pytel et al., the participants included were those diagnosed with PPA, rather than with bvFTD. In this study, the objective is to determine the effects of rTMS with personalized targeting on language and other measures including apathy levels. Compared to the PPA cohort, the staggering incidence of apathy in bvFTD makes it crucial to study the potential beneficial effects of rTMS on apathy in this population. A small (*n* = 9) open-label, before-and-after study on FTD in which the majority of participants had bvFTD and were treated with 10 sessions of HF rTMS over bilateral DLPFC showed improvement in the Montreal Cognitive assessment, letter and digit cancelation tests, Stroop reading time and error number, and Frontal Behavioral Inventory caregiver’s impression of daily functioning (75). However, this study did not assess the impact of rTMS on apathy scores. Overall, we encourage future research on the effects of rTMS to also focus on apathy, a common symptom linked with poor outcomes, particularly in individuals with bvFTD.

One notable and prevalent challenge in clinical practice is distinguishing apathy and depression (76). Apathy differs from depression in that depression generally impacts emotion, whereas apathy predominantly impacts volition (76). While neuroimaging and biomarker studies have detected significant distinctions between apathy and depression, prior research indicates that these two conditions exhibit comparable neurobiological mechanisms, such as Aβ pathology and vascular disease (77). We postulate that depression may have an impact on the relationship between rTMS and apathy based on the similarities between apathy and depression in various populations and the well-established antidepressant effects of rTMS. In this review, the included studies did not sufficiently consider the potential for the antidepressant effects of rTMS to influence the observed effects on apathy. Therefore, we recommend that future investigations shall include the conduct of multivariable analyzes to carefully explore the potential confounding effect of the change in depression levels between baseline and posttreatment on the relationship between rTMS effects and apathy and to determine if rTMS effects are independently associated with improvement of apathy.

The rating tools used to measure apathy in the included studies varied significantly which limits comparison of results across the studies. Various apathy scales are utilized in clinical and research environments, each exhibiting unique psychometric characteristics. In a previous systematic review of measurement properties of apathy tools that involved 57 studies, AES and LARS were recommended for measuring apathy in older adults and people living with dementia due to sufficient content validity, reliability, construct validity, structural validity and internal consistency (78). In PD patients, various apathy tools, including LARS, AES, AS, and SAS, were utilized; however, there is no consensus on which should be utilized in clinical trials (79, 80). Generally, there is no universally recognized gold standard for assessing apathy across different neuropsychiatric disorders. While robust psychometric properties hold significance, it is crucial to take into account additional factors when determining the most suitable apathy rating instrument, such as the type of scale (i.e., general scale or disease−/symptom-specific), specific apathy domains to be measured, the instrument’s sensitivity to detect changes within the required timeframe for treatment effects, information source/rater type, and setting (81). Furthermore, current literature also recognizes that apathy is not a single construct and may involve not only behavior, cognition and emotions, but also social interactions (82). Assessing apathy using validated scales is essential due to the diverse range of symptoms observed across several domains and the utilization of multiple measurement scales. A number of apathy scales now in use lack the evaluation of social apathy and a scale that encompasses crucial domains of apathy, including the social domain, would be beneficial. An instrument that also assesses social apathy is the Apathy-Motivation Index (AMI), which is a novel, brief, self- and caregiver-reported tool developed to assess behavioral, emotional and social facets of apathy, and was validated using other established rating scales (83, 84). We recommend that future research utilize instruments that address every dimension of apathy and are validated in various neurocognitive disorders in order to precisely define the impact of treatments on this complex symptom.

The presence of apathy has been associated with diverse dysfunctions in brain circuitry that vary depending on the specific condition. Apathy is typically distinguished by a decrease in activity primarily within prefrontal network (82). Aside from the prefrontal circuits, several neurophysiological and neuroimaging research studies demonstrated that apathy had been linked to complex aberrant processes in other superficial and deep brain regions including orbitofrontal cortex, medial prefrontal cortex, anterior/posterior cingulate gyrus, supplementary motor area, inferior temporal cortex, lateral parietal cortex, and striatum, manifested in various neuropsychiatric conditions, such as in AD/MCI and PD populations (85–90). A research investigation was conducted on a heterogeneous PPA cohort which revealed an association between reduced gray matter intensity in the right DLPFC and the manifestation of apathy (91). Post-stroke apathy arises as a consequence of infarction and ischemia that result in disruption to the fundamental brain regions and networks responsible for goal-directed behavior, such as involving the prefrontal cortex and basal ganglia (13). For TBI, the anatomical basis of apathy has received very little investigation, but lesions in certain neural structures were implicated, including the basal ganglia, medial frontal cortex, anterior cingulate gyrus, supplementary motor gyrus and hippocampus (92).

The precise mechanism by which rTMS affects apathy remains unclear. We speculate that the underlying mechanism by which rTMS influences apathy may involve alterations in the overall connectivity of the brain, which in turn may lead to a beneficial alleviation of apathy symptoms. Previous studies showed that the utilization of rTMS targeting the DLPFC had been observed to modulate the release of endogenous dopamine in various brain regions, including the mesostriatal and mesolimbic areas, as well as the anterior cingulate cortex and orbitofrontal cortex (93–95). Additionally, this intervention was theorized to enhance neuronal activity within the prefrontal cortex, induce synaptic plasticity mechanisms through long-term potentiation and long-term depression, promote neurotrophic effects on dendritic growth and sprouting, and exhibit neuroprotective properties (41, 96, 97). It was found that rTMS stimulation of the prefrontal cortex results in the increased release of dopamine in the caudate nucleus (98, 99). The administration of methylphenidate in rats and monkeys resulted in an elevation of dopamine release in the DLPFC (100, 101). It was considered that the potential resemblance in the mechanisms of action between methylphenidate and rTMS in their ability to modulate the dopamine activity in the prefrontal cortex represents a common driving process that results in improvement in apathy (41). Furthermore, rTMS may induce neuroplastic changes not only in the stimulated area but also in the associated cortical and subcortical regions (42). This is consistent with research on rTMS in post-stroke patients and suggests that rTMS can modify neural connectivity between hemispheres and have effects beyond the stimulation site (102). Additionally, the observed effects of rTMS after treatment are believed to be based on the overall network remodeling of the brain rather than solely on changes in individual motor-related regions (103). However, in cases where there are irreversible damage or severe disruption of connectivity pathways associated with the damaged area, the effects of stimulation on a specific region may be limited (42).

The careful consideration of the site of stimulation for rTMS holds significant importance. A significant number of the included studies opted to utilize HF rTMS specifically targeting the left DLPFC. This choice is supported by a substantial body of literature that has established a robust level of evidence for the efficacy of this approach, notably in the treatment of depression (104). It is thought that this effect on brain areas responsible for depression also extends to other regions associated with apathy through remote effects. On the other hand, Sasaki et al. preferred the medial prefrontal cortex (mPFC)/dorsal anterior cingulate cortex (dACC) as the stimulation site (43). The reasons for this choice were as follows: (1) defining the target site as the mPFC based on the pathophysiology of apathy (105), and (2) setting the stimulation intensity to the maximum value that a double-cone coil can deliver while ensuring safety by avoiding pain on the brain surface. A limited number of included studies in this review have focused on exploring stimulation of other cortical areas beyond those traditionally associated with apathy, driven by various hypotheses regarding the underlying mechanisms of this condition. Overall, it is imperative to conduct additional research that establishes a correlation between the impact of rTMS on different brain regions, as observed through functional imaging, and the ensuing changes in apathy in various neuropsychiatric conditions. Furthermore, given the existence of distinct domains of apathy (i.e., behavioral, cognitive, and affective), it is important to consider how the pathophysiology of the various conditions may selectively impact these domains. This consideration has significant implications in the research setting for determining the most appropriate stimulation sites for the rTMS intervention.

There are several methodological concerns present in the included studies that warrant recognition, as they pose a potential threat to the internal validity of their findings. Nearly all of the clinical trials included indicated that randomization was implemented; however, they overlooked to specify the precise method of randomization, including the allocation concealment technique, employed in the trials. Fortunately, the majority of studies included exhibited a similarity of the baseline characteristics among the treatment arms which limits potential confounding variable effects (i.e., indicating the success of randomization); however, as mentioned earlier, the included studies did not take into consideration the possible confounding effects of change in depression levels on the link between rTMS and apathy. In addition, several trials implemented blinding techniques for participants, yet they often overlook the masking of study personnel/rTMS technicians, thereby elevating the risk of performance bias. We recognize the difficulty in implementing the blinding of rTMS technicians as these would require the utilization of a blinded sham-coil that should also be visually and auditory similar to the blinded active-coil. Nevertheless, it deserves mentioning that due to the absence of anticipation in the control group, unblinding of participants/personnel can result in differential behaviors in the treatment arms (e.g., differential drop-outs) (106). In trials that necessitate prolonged and frequent administration of an intervention such as rTMS, ensuring the integrity of blinding is paramount to mitigate participant attrition. Moreover, the majority of trials successfully implemented the blinding of outcome assessors. This is a crucial strategy because most apathy scales, which are deemed objective measures due to the numerical conversion of included items, may contain items that are subjectively graded by the assessors. In addition to the acknowledged bias, other factors that diminish the certainty of the evidence of the reported effect estimates include small sample sizes of the studies contributing to the imprecision of effect estimates and inconsistencies in the conclusions across the studies. In studies that employed the single-group, before-after designs, interpretations of results are constrained due to the lack of an appropriate control group.

Overall, the comparability across studies in the included studies and the aggregation of relevant effect estimates are hindered by the variability in population characteristics, differences in the rTMS protocols and control groups, diverse outcome scales used to assess apathy and measures of treatment effect, and methodological disparities. The presence of these substantial variabilities hinders our capacity to provide precise recommendations regarding the most effective and optimized rTMS parameters to address apathy for each population discussed in this review. Therefore, for future studies, it is imperative to establish a suitable correlation between the underlying pathophysiology of apathy, the site of stimulation, and the patterns of stimulation.

Our review proposes the potential feasibility of rTMS as a therapeutic intervention for apathy despite the recognized limitations. Current limited evidence indicates that the use of rTMS holds promise in modulating the apathy signal in individuals with AD, PPA, MCI, and chronic stroke, but possibly less so in PD and mild TBI. Considering the acknowledged limitations of the included studies, it is essential for future research endeavors to prioritize the conduct of clinical trials with more robust designs aimed at confirming or refuting the preliminary findings presented in this review.

## Data availability statement

The original contributions presented in the study are included in the article/supplementary material, further inquiries can be directed to the corresponding author.

## Author contributions

AE: Conceptualization, Data curation, Formal analysis, Investigation, Methodology, Project administration, Resources, Software, Supervision, Validation, Visualization, Writing – original draft, Writing – review & editing. TH: Conceptualization, Data curation, Formal analysis, Investigation, Methodology, Validation, Writing – original draft, Writing – review & editing. JT: Data curation, Formal analysis, Investigation, Project administration, Software, Validation, Writing – original draft, Writing – review & editing. MB: Conceptualization, Data curation, Formal analysis, Investigation, Methodology, Project administration, Supervision, Validation, Writing – original draft, Writing – review & editing. AB: Conceptualization, Data curation, Formal analysis, Investigation, Methodology, Project administration, Resources, Supervision, Validation, Writing – original draft, Writing – review & editing.
